# Relating Lexical and Syntactic Knowledge to Academic English Listening: The Importance of Construct Representation

**DOI:** 10.3389/fpsyg.2020.00494

**Published:** 2020-03-31

**Authors:** Hongwen Cai

**Affiliations:** Center for Linguistics and Applied Linguistics, Guangdong University of Foreign Studies, Guangzhou, China

**Keywords:** lexical knowledge, syntactic knowledge, academic English, second language listening, construct

## Abstract

This study aims to resolve contradictory conclusions on the relative importance of lexical and syntactic knowledge in second language (L2) listening with evidence from academic English. It was hypothesized that when lexical and syntactic knowledge is measured in auditory receptive tasks contextualized in natural discourse, the measures will be more relevant to L2 listening, so that both lexical and syntactic knowledge will have unique contributions to L2 listening. To test this hypothesis, a quantitative study was designed, in which lexical and syntactic knowledge was measured via partial dictation, an auditory receptive task contextualized in a discourse context. Academic English listening was measured via a retired IELTS listening test. A group of 258 college-level native Chinese learners of English completed these tasks. Pearson correlations showed that both lexical and syntactic measures correlated strongly with English listening (*r* = 0.77 and *r* = 0.67 respectively). Hierarchical regression analyses showed that both measures jointly explained 62% of the variance in the listening score and that each measure had its unique contribution. These results demonstrated the importance of considering construct representation substantially and using measures that well reflect constructs in practical research.

## Introduction

It is not uncommon for researchers to report different or even contradictory findings when they try to address the same issue in second language (L2) studies. A case in point is the relative importance of lexical and syntactic knowledge in L2 listening comprehension, where mixed findings have been reported, some alluding to the sole significance of lexical knowledge while downplaying or masking the role of syntactic knowledge (Mecartty, 2000; Stæhr, 2009; Vandergrift and Baker, 2015; Cheng and Matthews, 2018; Matthews, 2018), others rendering the relative importance unclear (Oh, 2016; Wang and Treffers-Daller, 2017) or resorting to the more general construct of linguistic knowledge and avoiding the distinction between lexical and syntactic knowledge (Andringa et al., 2012).

The different findings and their relative generalizability may be attributed to various factors, such as the characteristics of the participant groups, the treatments delivered to the participants, the properties of the instruments used, and the settings of the studies. Among these factors, the instruments are of vital importance to the construct validity of the studies (Shadish et al., 2002; Shadish, 2010). In the case of lexical and syntactic knowledge, the mixed findings may be attributed, at least partially, to the variety of instruments used in different studies, which are based on different theoretical underpinnings and construct representations. It is, therefore, important to understand the construct definitions and specifications related to the various instruments before the contradictions between findings can be resolved.

Following this reasoning, the relevant studies will be reviewed to compare the various construct representations of lexical and syntactic knowledge under a uniform framework, with a view to identifying the key features that are central to L2 listening. On the basis of these key features, an observational study will be designed in the academic English context to quantify the relative importance of lexical and syntactic knowledge in L2 listening, with a view to resolving the contradictions between findings from earlier studies.

### Literature Review

#### Lexical and Syntactic Processes in Listening

To establish a uniform framework for comparing the construct representations of lexical and syntactic knowledge, a brief account of psycholinguistic theories of language comprehension is inevitable. Fortunately, descriptions of the key stages of comprehension are more or less the same across the rich variations of models, such that a “basic” model can be conceptualized, comprising word-, sentence-, and discourse-level processes (Fernández and Cairns, 2018). A variation of this basic model often cited in applied linguistics literature is the three-stage cognitive model of Anderson (2015), consisting of *perception*, *parsing*, and *utilization*. The division into three stages is supported by neurological evidence, such that psychologists are able to identify the different combinations of brain regions involved in the three stages (Anderson, 2015).

In the L2 listening literature, the three stages are sometimes rephrased as *decoding*, *parsing*, and *meaning construction* (Field, 2011). In brief, the listener converts the acoustic-phonetic signal into words, relates the words syntactically for a combined meaning, and enriches the meaning by integrating it with meaning derived from earlier text, context, and background. While the three-stage model deals with the cognitive processes of listening comprehension, these processes depend upon a multitude of sources, linguistic, contextual, and schematic, among which linguistic sources can be further classified into phonetic, phonological, prosodic, lexical, syntactic, semantic, and pragmatic processes (Lynch, 2010).

The interplay between lexical and syntactic processes is an essential part of the cognitive processes in L2 listening. For one thing, word-level processes, such as the identification of a single word, depend on both lexical-semantic and syntactic cues in the context (Buck, 1991, 1994; Anderson, 2015). Neurologically, the speech signal of a word needs to be combined with information about its acoustic-phonological, syntactic, and conceptual semantic properties before it is recognized (Hagoort, 2013). Similarly, parsing also draws on both syntactic and lexical-semantic cues (Anderson, 2015). Underlying this process are two classes of neural mechanisms—lower-order bottom–up mechanisms that enable the lexical-semantic and morphosyntactic categorizations of the speech input and higher-order bottom-up and predictive top–down mechanisms that assign the complex relations between the elements detected in a sentence and integrate them into a conceptual whole (Skeide and Friederici, 2018). There is also evidence that the lexical-semantic and morphosyntactic categorizations are parallel processes, as they occur within 50–80 and 49–90 ms, respectively, after the onset of the speech signal (Friederici, 2012). In general, the three stages of listening comprehension are described as partly parallel and partly overlapping (Anderson, 2015).

#### Findings in L2 Listening Research

Findings in L2 listening research have mirrored the interplay between lexical and syntactic processes, though to different degrees. For example, some studies on L2 English and French listening focused solely on the correlation between lexical knowledge and L2 listening (Stæhr, 2009; Vandergrift and Baker, 2015; Cheng and Matthews, 2018; Matthews, 2018), reporting significant correlations between 0.39 and 0.73. With regard to the psycholinguistic theories reviewed above, the emphasis on lexical knowledge may have masked the contribution of syntactic knowledge to L2 listening. For the purpose of this study, however, these findings can be regarded as an initial indication of how strong the correlation between lexical knowledge and L2 listening can be.

That being said, the wide range of correlation estimates from these studies points to a potential problem—the inconsistent measures of the same construct. In fact, Cheng and Matthews (2018) deliberately compared the correlations of three different measures of lexical knowledge to L2 English listening and found that the correlations ranged between 0.39 and 0.71. The measure of lexical knowledge may also be confounded with other measures. In the study of Wang and Treffers-Daller (2017) on L2 English listening, the measures included a general language proficiency test, a vocabulary size test, and a questionnaire of metacognitive awareness. However, the general language proficiency test included a large number of items targeting lexical knowledge. Although their results of hierarchical regression analyses showed that general language proficiency and vocabulary both contributed uniquely to the variance of listening, the size of these contributions is subject to this confounding effect.

Another problem arises in empirical studies when the masking of syntactic knowledge in L2 listening is so conspicuous that it may negate the interplay between lexical and syntactic processes. Mecartty’s (2000) study on L2 Spanish learners found that both lexical and syntactic knowledge were significantly correlated with L2 listening (*r* = 0.38 and *r* = 0.26 respectively), but his hierarchical regression analysis showed that only lexical knowledge explained 13% of the variance in listening. Although the addition of syntactic knowledge to the model increased the percentage of explained variance to 14%, the *R*^2^ change was not statistically significant, and Mecartty concluded that syntactic knowledge had no unique contribution, which contradicts the psycholinguistic theories that both syntactic and lexical-semantic cues are necessary for listening comprehension. Interestingly, the correlation between lexical and syntactic measures in Mecartty’s (2000) study was estimated at *r* = 0.34, which, though significant, could be considered weak. This practically rules out the possibility of substantial overlap between the two measures being the cause of the insignificant *R*^2^ change.

Other studies that were related to the contribution of lexical and syntactic knowledge to L2 listening yielded findings that agreed more with psycholinguistic theories. A common methodological feature among these studies is that L2 listening was regressed on multiple independent variables. Oh’s (2016) study on L2 English listening included four measures of processing speed, two measures of grammar, and three measures of vocabulary. While she found significant correlations between listening and all but one of the processing speed measures, she reported that none of the three groups of measures explained a unique portion of variance in listening when the other two groups of measures were already entered into the hierarchical regression model, which seems to suggest that while lexical and syntactic knowledge both contribute to listening, they were not distinct processes. The assumption of joint contribution of lexical and syntactic knowledge agreed with psycholinguistic theories, but the lack of distinction between the two processes may be considered as construct confounding for the purpose of this study.

Among the studies published so far, Andringa et al. (2012) have captured the psycholinguistic sophistication of L2 listening most faithfully. These authors constructed a structural equation model to explain L2 Dutch listening with a multitude of variables, including three measures of linguistic knowledge, five measures of processing speed, and six cognitive measures of intelligence. They found that the latent construct of linguistic knowledge indicated by vocabulary, grammatical processing, and segmentation (of speech stream into words) explained 90% of the variance in listening. As no distinction was made between lexical and syntactic knowledge in their original model, this result cannot be compared to those discussed above. For this purpose, a hierarchical multiple regression was run by the author of this paper on the R package “lavaan” version 0.6-2 (Rosseel, 2012), using the correlation matrix and standard deviations reported in the original paper in lieu of raw data. The *R*^2^ was estimated at 0.46 when L2 listening was regressed on vocabulary only and at 0.59 on grammar only, but increased to 0.67 when both predictors were entered. This result was closest to psycholinguistic findings in that lexical and syntactic sources both had unique contributions to the variance in L2 listening, and that the joint contribution of the two sources had significantly stronger explanatory power than single sources. Moreover, the lexical and syntactic measures were moderately correlated with each other (*r* = 0.60), which ruled out the threat of multicollinearity.

#### The Importance of Measures

With regard to the relative importance of lexical and syntactic knowledge in L2 listening, the most notable contradiction was between the findings of Andringa et al. (2012) and those reported by Mecartty (2000). Andringa et al. (2012) themselves noted that linguistic knowledge explained a larger percentage of variance in their study than in Mecartty’s (2000) study. This is an important observation, in that 90% was considerably greater than 14%, which merits much further investigation. A comprehensive search for possible reasons may cover experimental factors or treatments, classificatory factors or personal variables, situational variables or settings, and outcome measures or observations (Shadish et al., 2002), as there are differences between the two studies in all these aspects. A heuristic search, however, could be based on the explanations of the authors themselves, who know the details of their study best. The first possible reason given by Andringa et al. (2012) was that measurement error had been cleared in the latent variable model they used, but even in raw score terms, lexical and syntactic knowledge explained 67% of variance in L2 listening, as this author’s reanalysis demonstrated. Another factor Andringa et al. (2012) postulated was restriction of range in L2 proficiency in Mecartty’s study. This could have attenuated correlations as well, but a closer examination of the coefficients of variation (CVs) yielded comparable results: 0.24 for L2 listening, 0.33 for lexical knowledge, and 0.15 for syntactic knowledge in Andringa et al. (2012) and 0.35 for L2 listening, 0.24 for lexical knowledge, and 0.25 for syntactic knowledge in Mecartty (2000). Calculated as the ratio of the standard deviation to the mean, the CV is a standardized measure of dispersion such that it can be directly compared between two studies. It follows that the comparable results can be taken as evidence that restriction of range was not a key factor that attenuated correlations in Mecartty’s study. Therefore, the more probable reason that underlies the different findings in the two studies may be that the linguistic knowledge tests in Andringa et al. (2012) were “more pertinent to listening,” whereas “grammatical knowledge was measured in a production task in Mecartty” (p. 70).

A more common term for pertinence is “construct relevance,” and the pertinence issue raised by Andringa et al. (2012) is essentially the issue of construct representation (Bachman, 1990), which takes the form of measures of L2 listening, lexical knowledge, and syntactic knowledge. Underlying the reasoning of Andringa et al. (2012) is the assumption of how lexical and syntactic knowledge should be measured when examining their role in L2 listening. Though the measure of L2 listening itself is also a construct representation issue of no less importance, this paper will be confined to the discussion of the independent variables. A closer examination of the above-mentioned reason reveals two basic conceptual dichotomies familiar to most researchers in applied linguistics, the dichotomy of visual and auditory modes and the dichotomy of receptive and productive skills. Take the syntactic measure used in Andringa et al. (2012); the underlying construct was knowledge of the “distributional and combinatorial properties” of the Dutch language, most notably word order and agreement. A judgment task was designed, which required the participants to judge whether a fragment presented aurally was a possible sentence-initial string in Dutch, e.g., *Die stad lijkt heel* (“That city seems very”) and *Precies ik weet* (“Exactly I know”). In comparison, Mecartty (2000) used two syntactic measures, the first of which was a sentence completion task aiming to measure “local-level understanding of the grammatical features” of Spanish and requiring the participants to complete Spanish sentences with function words, such as *Me gusta aquel automóvil; _____ me gusta el rojo* (“I like that car; I ____ like the red one”). The second task was grammaticality judgment and error correction, aiming to measure knowledge of the “underlying rules” of Spanish, which required the participants to identify grammatical errors in Spanish sentences and correct them, such as ^∗^*Compró el carro y transportó lo a su garaje* (“He bought *the* car and transported it to his garage”). In terms of the two dichotomies, the syntactic measure used in Andringa et al. (2012) was an auditory receptive task, whereas Mecartty’s (2000) syntactic measures were visual productive tasks. As listening is an auditory receptive language use activity, it is natural to expect the former to be more strongly correlated with listening than the latter. More specifically, difficulty in a productive task does not necessarily transfer to a receptive task. For example, an L2 Spanish learner may have difficulty in choosing the right word to complete the sentence *Me gusta aquel automóvil; _____ me gusta el rojo*, but no difficulty at all in understanding the sentence presented in auditory mode, even if the incomplete sentence is presented. In contrast, identifying *Die stad lijkt heel* as a sentence-initial string in Dutch is helpful for understanding the meaning of the whole sentence containing the string, as word order is important in Dutch syntax (Oosterhoff, 2015) and thus a key factor for parsing (Anderson, 2015). In sum, a relevant measure of syntactic knowledge in L2 listening should take the form of an auditory receptive task with a focus on the key processes in parsing.

The same features apply to relevant measures of lexical knowledge in L2 listening, as evidenced by the three measures of lexical knowledge in Cheng and Matthews (2018). Intended for receptive vocabulary, their first measure took a multiple-choice format after the Vocabulary Levels Test of Nation (2001). Their second measure, targeting productive vocabulary, was adapted from the controlled-production vocabulary levels test of Laufer and Nation (1999) and required the participants to complete a sentence with the target word, whose initial letters were provided. Both measures were presented visually. The third measure of receptive^1^ vocabulary took the form of a partial dictation task and required the participants to complete each sentence they heard with a missing word. All three measures covered the first 5,000 frequency levels of word lists extracted from the British National Corpus (BNC, Leech et al., 2001). The researchers correlated these measures with the scores from a retired IELTS listening test and estimated Pearson correlation at 0.39 for the visual receptive measure, 0.55 for the visual productive measure, and 0.71 for the auditory receptive measure. This is evidence that auditory receptive measures of lexical knowledge are most relevant to L2 listening, due to similarity in task characteristics between the lexical measure and the L2 listening test. Another dimension that may have contributed to the relevance of lexical measures may be the context provided. The visual productive measure in Cheng and Matthews (2018) was contextualized in single sentences, whereas the visual receptive measure was decontextualized, which may explain why the former was more strongly correlated with L2 listening (*r* = 0.55) than the latter (*r* = 0.39). A similar pattern is uncovered when comparing the correlation with L2 listening of the sentence-based visual receptive measure in Andringa et al. (2012) and the correlation with L2 listening of the decontextualized visual receptive measure in Mecartty (2000). Correlation was higher when lexical knowledge was measured in sentential context (*r* = 0.68) but lower when the measure was decontextualized (*r* = 0.34).

In sum, construct representation is a key factor that affects the findings on the relative importance of lexical and syntactic knowledge in L2 listening. Different measures of lexical and syntactic knowledge may represent different features of the constructs, which affects their relevance to L2 listening. More specifically, the visual/auditory, receptive/productive, and contextualized/decontextualized dichotomies may be key considerations for examining the contribution of lexical and syntactic knowledge to L2 listening.

### Research Questions

To examine the above understanding, and to demonstrate the importance of theoretical underpinnings in practical research, the findings of Andringa et al. (2012) and Cheng and Matthews (2018) need to be replicated, with regard to the relationship between lexical and syntactic knowledge and L2 listening. Following the relevance principle, it is hypothesized that when lexical and syntactic knowledge is measured in auditory receptive tasks contextualized in natural discourse, the measures will be more relevant to L2 listening, so that both lexical and syntactic knowledge will have unique contributions to L2 listening. To test this hypothesis, the replication study should include both lexical and syntactic measures, similar to Andringa et al. (2012), but will be set in the academic English context, similar to Cheng and Matthews (2018). Two key research questions (RQs) are:

(1) How do lexical and syntactic knowledge correlate with L2 listening in the academic English context?
(2) Do lexical knowledge and syntactic knowledge have unique contributions to L2 listening in the academic English context?

RQ1 aims to measure the degree of association between lexical and syntactic knowledge and L2 listening. It is hypothesized that with a high level of relevance, Pearson correlations around 0.70 may be expected for both measures, similar to the findings with regard to the sentence-based visual receptive measure in Andringa et al. (2012) and the auditory receptive measure in Cheng and Matthews (2018). RQ2 is based on the psycholinguistic theories reviewed above, assuming that lexical and syntactic processes are distinct but contribute jointly to listening. It is hypothesized that both lexical knowledge and syntactic knowledge have unique contributions to L2 listening, and that the joint contribution of the two sources has stronger explanatory power.

## Materials and Methods

### Participants

The study was conducted on 258 native Chinese learners of academic English as a second language. At the time of the study, they were first-year English majors enrolled in a university in China. Their mean raw score on the academic English listening test used in this study (15.33) converted to a band score (5) according to the official conversion table^2^ close to the mean band score (5.89) on IELTS listening of test-takers from China in 2018^3^.

### Instruments

The measure of L2 academic English listening was a retired IELTS listening test published by Cambridge University Press. No participants had had access to the material prior to this study. The input material included the recordings of two monologs and two conversations, with 40 printed questions in four different formats—multiple-choice questions with four options, matching questions, judgment questions with three options (yes/no/not given or true/false/not given), and fill-in-the-blank questions in the form of questionnaires or forms to be filled. The monologs and conversations were recorded by native English speakers and were set in a variety of everyday social and educational/training contexts. These were designed to measure the ability to understand the main ideas and detailed factual information, the opinions and attitudes of speakers, and the purpose of an utterance and the development of ideas^4^.

The measures of lexical and syntactic knowledge were integrated into a partial dictation task. Eight minutes of recording of the IELTS listening test were selected as the auditory input of the partial dictation, so that the same level of naturalness in spoken English can be achieved (Cai, 2013). The selection was based on the requirement that at least 10 words could be found in the recording on each of the three frequency-based levels, i.e., the 1,001–2,000 frequency range, the 2,001–3,000 frequency range, and the 3,001–5,000 frequency range, of the BNC (Leech et al., 2001). This decision was based on the findings of Matthews (2018) that each of these three levels had unique contributions to L2 listening performance, and on the practice to include 10 items from each 1,000-word-family level for testing vocabulary size (Nation and Beglar, 2007). Each blank was produced by taking away a single word or a two-to-three-word phrase. To give the participant sufficient time to write down the words and phrases they heard, the blanks were set apart at intervals of at least nine words, as the underlined segments (17, 18, and 19) in the following excerpt exemplify.

… *I’d like to say at this point that you shouldn’t worry (17) if this process doesn’t work all that quickly – I mean occasionally there are postal problems, but most often the (18) hold-up is caused by references – the people you give as (19) referees, shall we say, take their time to reply.*

The interval between blanks No. 18 and No. 19, which both involved single words, was the minimum nine words. The interval between a blank for a missing phrase and another blank was typically longer to allow more time for writing. For example, the interval between blanks No. 17 and No. 18 in the above excerpt was 17 words. This excerpt also exemplifies the items included in the lexical and syntactic scales. The lexical scale was made up of 30 single words, 10 from each of the three levels described above. For example, the words “referees” (blank No. 19) and “hold-up” (blank No. 18) were from the 2,001–3,000 and 3,000–5,000 levels respectively. Each correctly spelled word was worth 1 point, so that the maximum score was 30 for the scale.

The syntactic scale consisted of 15 two-to-three-word phrases, such as “if this process” for blank No. 17, which is the initial string of a subordinate clause, consisting of the subordinating conjunction “if” and the noun phrase “this process,” which serves as the subject of the clause. Identifying this phrase involves knowledge of word order and subordination, which are both important syntactic cues for parsing (Anderson, 2015). The other syntactic features involved in the items included ellipses, noun conjunctions, pronouns, parentheses, emphatic expressions, etc. (see Appendix for details.) To avoid confounding with lexical processes, none of the phrases in the syntactic scale included words beyond the first 1,000-word-family level of the BNC (Leech et al., 2001). As word order is the key syntactic feature that influences parsing in English (Anderson, 2015), the participants’ responses were scored according to the degree of conformity to the original word order. The maximum score for each segment was 2, for responses that retrieved the original phrase in its full form, for example, “if this process.” A score of 1 was given to responses that retrieved only a semantically proper pairwise sequence, e.g., “this process,” Otherwise the response would be given a score of 0, regardless of the number of words retrieved, e.g., “if process” or “process.” To avoid inconsistent judgments, misspelt words were considered errors. The maximum score for each of the 15 segments was 2 points, and the maximum score for the full scale was 30.

As the lexical and syntactic measures both took the form of a partial dictation task, word recognition may be the key process underlying both measures, which poses a major threat to the validity of the syntactic measure. For preliminary evidence of validity, a homogeneity test by way of internal consistency (Anastasi and Urbina, 1997; Urbina, 2014) was conducted. The lexical scale was broken into three subscales, each consisting of 10 items from each of the three levels described above, i.e., the 1,001–2,000 frequency range, the 2,001–3,000 frequency range, and the 3,001–5,000 frequency range, of the BNC (Leech et al., 2001). Coefficient alpha was calculated at 0.85 for the three subscales (which coincided with the item-level estimate reported in Table 1) but dropped to 0.78 when the syntactic measure was included as the fourth subscale. As internal consistency is essentially a measure of homogeneity (Anastasi and Urbina, 1997; Urbina, 2014), this is evidence that the three lexical subscales constituted a more homogeneous scale, whereas the syntactic measure was more heterogeneous to the lexical measure. Together with the content analysis presented above and detailed in the Appendix, this provides the preliminary evidence for interpreting the 15 phrases as a syntactic measure.

**TABLE 1:** Descriptive statistics, reliabilities, and correlations (*n* = 258).

	Mean	*SD*	Maximum	Minimum	Skewness	Kurtosis	Alpha	Correlation* (95% CI)	
								Lexical	Syntactic
Listening	15.33	5.20	29	5	0.55	−0.16	0.78	0.77 (0.71,0.81)	0.67 (0.60,0.73)
Lexical	9.38	5.07	25	1	0.78	0.42	0.85		0.70 (0.64,0.76)
Syntactic	12.05	5.00	25	1	0.34	−0.30	0.72		

*All correlations were significant at the 0.001 level.

### Data Collection Procedures

The IELTS listening test was administered in its paper-and-pen form in a computerized language lab as part of a mid-term test for the academic listening course. In accordance with the official IELTS administration procedures, the participants heard the recordings once only and responded to the questions in 30 min, after which they transferred their responses to a commercial web-based testing platform, which saved the responses as a downloadable Microsoft Excel file for scoring.

The partial dictation task was completed immediately after the participants submitted their listening test responses online, as another part of the mid-term test. The task was also administered in its paper-and-pen form. The participants heard the recordings once only, after which the participants submitted their responses to the same testing platform. The responses were also downloaded as a Microsoft Excel file for scoring.

### Data Analysis

The scores used in the analyses were numbers of correct responses. The maximum score was 40 for the listening test and 30 for the lexical and syntactic scales. To answer RQ1, scores on the lexical and syntactic scales were correlated to the score on the listening test. To answer RQ2, the listening score was regressed on the lexical and syntactic scales in two sequential analyses. The first analysis started with the lexical scale in the first step, with the addition of the syntactic scale in the second step. The second analysis was conducted in the reverse order, starting with the syntactic scale. All analyses were conducted in SPSS18.

## Results

### Correlations

Correlations between lexical and syntactic measures and L2 academic English listening were calculated to answer RQ1. Table 1 reports the mean, standard deviation, and internal consistency reliability (coefficient alpha) for each of the three measures, as well as Pearson correlations between each pair of measures with their 95% confidence intervals.

Prior to discussing the descriptive statistics, the internal consistency reliability of the three scores should be examined. Coefficient alpha was estimated at 0.78 for the listening score, 0.85 for the lexical score, and 0.72 for the syntactical score. These were considered acceptable for the study. The coefficient of variation can be calculated for each measure from the mean and standard deviation reported in Table 1, i.e., 5.20/15.33 = 0.34 for listening, 5.07/9.38 = 0.54 for the lexical score, and 5.00/12.05 = 0.41 for the syntactical score. The CV for the listening score was comparable to the estimates calculated from the descriptive statistics reported in Mecartty (2000) and Andringa et al. (2012). However, the CVs for the lexical and syntactical scores were considerably greater than those calculated from the two previous studies. Taken together, these were evidence that restriction of range in the three scores did not attenuate the correlations seriously. The skewness and kurtosis estimates of the three scores are also reported in Table 1. None of these had an absolute value greater than 1, so the scores were considered to be approximately normally distributed, which supported the use of Pearson correlations to represent the bivariate relationships.

As Table 1 shows, the three pairwise correlations were all close to 0.70, comparable to findings reported in Andringa et al. (2012) and Cheng and Matthews (2018). Considered separately, both lexical and syntactic scores were moderately correlated with the L2 listening score. The correlation between lexical and syntactic scores was also moderate, consistent with psycholinguistic theories that lexical and syntactic processes are distinct processes in listening.

### Regression Analyses

**FIGURE 1:**
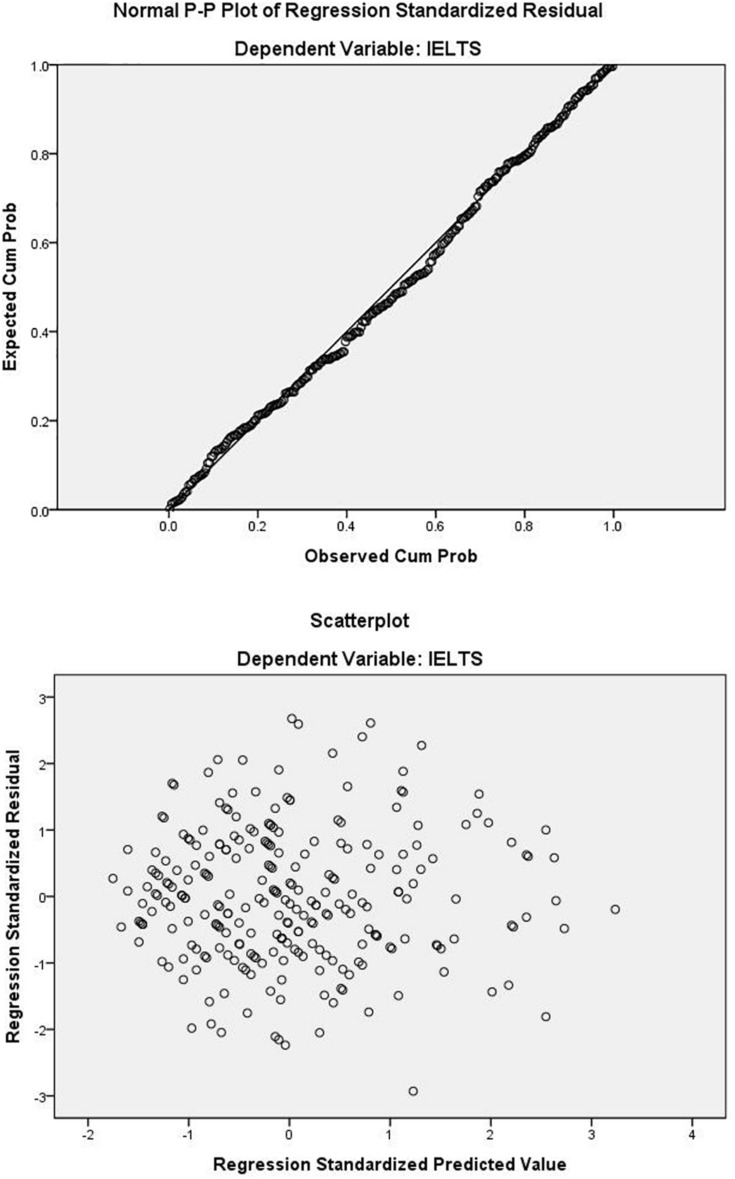
Regression diagnostics.

To answer RQ2, two hierarchical regression analyses were conducted, both regressing L2 academic English listening on the lexical and syntactic scores, but with different predictors in each step. Prior to the analyses, the outlier and collinearity assumptions were examined. The maximum value of Cook’s distance in the sample was 0.055, far less than the critical value of 1, indicating that there were no overly influential cases that warranted exclusion from the analyses (Cook, 1977). The tolerance was estimated at 0.504, indicating that around half of the variance in one predictor could be explained by the other predictor. The corresponding variance inflation factor was 1.984, and multicollinearity was not considered a serious threat to result interpretation. After the regression analyses, diagnostics were also run to examine the normality and homoscedasticity of the residuals. Figure 1 displays the resulting plots.

The upper panel is the normal P-P plot of the standardized residuals from the regression model, which displays only minor deviations from normality. The lower panel is the scatterplot with the standardized predicted value on the X-axis and the standardized residuals on the Y-axis. No obvious deviation from homoscedasticity is observed. Therefore, the two regression analyses were considered appropriate.

In the first analysis, the lexical score was entered as a sole predictor of L2 academic English listening in the first step, with the addition of the syntactic score in the second step. The regression with only the lexical score was significant, *R*^2^ = 0.59, adjusted *R*^2^ = 0.59, *F*(1,256) = 369.76, *p* < 0.001. The addition of the syntactic score produced a significant *R*^2^ change, *R*^2^ = 0.03, *F*(1,255) = 22.24, *p* < 0.001. These results showed that lexical score alone was a good predictor of L2 academic English listening, explaining 59% of the variance in the listening score. The addition of the syntactic score contributed 3% more to the variance in the listening score.

The second analysis reversed the order and started with the syntactic score as a sole predictor of L2 academic English listening, with the addition of the lexical score in the second step. The regression with only the syntactic score was significant, *R*^2^ = 0.45, adjusted *R*^2^ = 0.45, *F*(1,256) = 208.46, *p* < 0.001. The addition of the lexical score produced a significant *R*^2^ change, *R*^2^ = 0.18, *F*(1,255) = 118.53, *p* < 0.001. These results showed that syntactic score alone was a good predictor of L2 academic English listening, explaining 45% of the variance in the listening score. The addition of the lexical score contributed 18% more to the variance. In either order, both predictors were able to account for 62% of the variance in the listening score.

In answer to RQ2, the above results show that both lexical and syntactic processes had unique contributions to L2 listening in the academic English context.

## Discussion

### Comparability to Earlier Studies

The correlation and regression analyses have yielded results that agree more with Andringa et al. (2012) and Cheng and Matthews (2018) than with Mecartty (2000). When considered separately, both lexical and syntactic measures correlated moderately with L2 academic English listening, with Pearson correlations close to 0.70. When considered jointly, both lexical and syntactic measures had unique contributions to the variance in the listening score. These results have confirmed the hypotheses stated earlier. More generally, they have provided evidence in support of the claim that different degrees of relevance in the measures will yield different results with regard to the relative importance of lexical and syntactic knowledge in L2 listening. More specifically, contextualized auditory receptive measures of lexical and syntactic knowledge are more similar to L2 listening tasks in terms of task characteristics and are considered more relevant to L2 listening in this sense, which explained the different results between Mecartty (2000) and Andringa et al. (2012). In particular, the lower correlations between lexical and syntactic measures and L2 listening in Mecartty (2000) may be attributed to the decontextualized visual feature of the lexical measure and the visual productive feature of the syntactic measure. The lack of unique contribution of syntactic knowledge to L2 listening in Mecartty (2000) may also be attributed to the same features.

It is also interesting to compare the findings of these studies to similar studies on L2 reading. Studies on the relative significance of lexical and syntactic knowledge in L2 reading have also yielded mixed results—some studies found a greater contribution of lexical knowledge (Bossers, 1992; Brisbois, 1995; Yamashita, 1999), while others reported heavier regression weights of syntactic knowledge (Shiotsu and Weir, 2007). Shiotsu and Weir (2007) emphasized the difference between a structural equation model and a regression model but also noted that sample size, test difficulty relative to the participants, characteristics of the participants, and the nature and reliabilities of the instruments used are important methodological factors that may explain the differences between studies. The commonality between the findings of the present study and those of Shiotsu and Weir (2007) is that both lexical and syntactic knowledge have a unique contribution to L2 English comprehension.

### Importance of Theoretical Underpinnings

The comparison of results between this study and earlier studies also demonstrates the importance of theoretical underpinnings in practical research. For example, the findings that syntactic knowledge does not contribute uniquely to the variance of L2 listening beyond lexical knowledge (Mecartty, 2000) are difficult to explain in light of psycholinguistic theories (Field, 2011; Anderson, 2015; Fernández and Cairns, 2018; Skeide and Friederici, 2018), whereas an emphasis on the joint contribution of lexical and syntactic knowledge (Andringa et al., 2012) agrees in principle with these theories and relative findings. This shows the importance of basing the measures on clear theoretical definitions of the constructs (Bachman, 1990).

The literature review has focused on psycholinguistic theories as the framework for depicting the partly parallel and partly overlapping relation between lexical and syntactic processes (Anderson, 2015). This coincides with findings in applied linguistics. For example, the verbal protocol studies of Buck (1991, 1994) found that L2 English listening tasks intended to test lexical knowledge turned out to involve higher-order processes, including syntactic processes. In turn, these findings also coincide with the lexico-grammatical approach to language studies in contemporary linguistics, which views lexis and syntax as the two ends of one continuum (Broccias, 2012; Sardinha, 2019). However, adopting a psycholinguistic framework offers the convenience of smooth transition to cognitive diagnostic assessment of listening, which is gaining increasing attention in L2 assessment (Lee and Sawaki, 2009; Aryadoust, 2018).

Another issue raised in the literature review is construct confounding, which reduces the relevance of results from Oh (2016) and Wang and Treffers-Daller (2017) to the issue under consideration in this study. The relative importance of lexical and syntactic processes in L2 listening was not distinguished in Oh’s (2016) results, while lexical knowledge was intertwined with general language proficiency in Wang and Treffers-Daller (2017). It is a pity that these studies do not provide further evidence for examining the theoretical relationship between lexical knowledge, syntactic knowledge, and L2 listening.

In passing, it is worthwhile to mention that the relationship between lexical knowledge, syntactic knowledge, and L2 listening is not only of theoretical significance but also has practical implications. In practice, L2 listening is often assessed as a uniform skill for general purposes such as placement, certification, progress monitoring, and teaching evaluation (Bachman and Palmer, 2010). However, there is a growing need for diagnostic assessment that calls for more fine-grained understanding of the cognitive processes that underlie L2 listening activities, which invariably include lexical and syntactic processes (Field, 2009, 2013; Goh and Aryadoust, 2014; Alderson et al., 2015; Harding et al., 2015).

### Generalizability Issues

Closely related to theoretical underpinnings is the idea of construct validity, which is a key requirement for making causal inferences in Campbell’s validity framework (Shadish et al., 2002; Shadish, 2010). One of the key reasons given by Andringa et al. (2012) to account for the differences between their results and Mecartty’s (2000) results was the different instruments used in the two studies. In a recent commentary, Schmitt et al. (2020) recommended argument-based approaches for vocabulary test development and validation, which “start with a clear and explicitly stated purpose and provide structured and comprehensive evidence for justifiable interpretations.” Earlier, Read (2000) emphasized the important role of context in a vocabulary test and argued against presenting words in isolation. It is the hope of this author that the present study provides some guidelines on how to suit the specific characteristics of assessment tasks (such as the visual/auditory, receptive/productive, and contextualized/decontextualized dichotomies) to the purpose for researchers who need a vocabulary test as an instrument in their future studies.

The other two reasons provided by Andringa et al. (2012) in explanation of the differences between their results and those of Mecartty (2000), i.e., measurement error and attenuated correlation due to restriction of range, were both issues related to the statistical validity of the studies in Campbell’s validity framework (Shadish et al., 2002; Shadish, 2010). While raw scores were used for replication purposes, restriction of range was not found to be a serious problem in this study. Together with relevance and theoretical underpinnings, both of which are construct validity issues in Campbell’s framework, they form the foundations for the generalizability of findings of this study. The measures of lexical and syntactic knowledge in this study were not exactly the same as those used in Andringa et al. (2012) and Cheng and Matthews (2018) but were comparable to them with regard to features of relevance. This means that if similar relevant measures are used in future studies, the researcher may expect to obtain similar results.

As for the measure of L2 academic English listening, this study has used the IELTS listening test, which was also used in Cheng and Matthews (2018), albeit not the same version. There is some threat to generalizability here, as the IELTS listening test has been criticized for underrepresenting the listening construct by tapping only the ability to understand explicitly stated information and to make paraphrases (Geranpayeh and Taylor, 2008; Field, 2009; Aryadoust, 2013). More generally, the construct definition of L2 listening, i.e., the dependent variable, has not been compared across earlier studies, as it was only vaguely mentioned in Andringa et al. (2012). Furthermore, the task characteristics of L2 listening have not been compared between earlier studies, or between this study and earlier studies. The visual/auditory, receptive/productive, and contextualized/decontextualized dichotomies have been proposed as the key features, but other task characteristics such as topical knowledge, linguistic complexity, speed, and response format also play a key role in the listening process (Bachman and Palmer, 2010; Taylor and Geranpayeh, 2011; Révész and Brunfaut, 2013). Therefore, comprehensive studies that address variations in both the independent and dependent variables, with clear definition and operationalization, will provide much insight into the issue under consideration in this study. For this study, the construct of L2 listening should be understood with these limitations in mind.

The findings of this study could have been more convincing if multiple types of measures had been used, so that direct comparison could be made between the visual/auditory, receptive/productive, and contextualized/decontextualized dichotomies, similar to what Cheng and Matthews (2018) did in their study. Furthermore, this study has used raw scores in regression models to enable comparison to Mecartty’s (2000) results, but latent variable models would promise more stable results with measurement errors considered, as Andringa et al. (2012) have done.

It was mentioned in the literature review that personal variables may also constitute a significant source of difference across studies. The participants of this study were more similar to those in Cheng and Matthews (2018) but were from a single major. In contrast, for example, the participants in Andringa et al. (2012) were adults with more varied ages, first language backgrounds, and socioeconomic statuses. These factors have been treated as random in the regression model but may play a systematic role. This is a pending question before a more comprehensive study is conducted.

Furthermore, the field is moving fast ahead, with new technologies being added to the repertoire of research methods. The psycholinguistic studies reviewed earlier have used event-related potential to capture neural activity related to both sensory and cognitive processes in listening (Friederici, 2012; Hagoort, 2013; Skeide and Friederici, 2018). Recently, there are also scholars who attempt to use eye tracking to unveil the listening process. For example, Aryadoust (2019) and Holzknecht (2019) found that test-takers spend much time on reading the test items and answering them, thus confusing listening ability with reading ability. These studies have both theoretical and methodological significance. Theoretically, they shed light on the cognitive process of L2 listening comprehension; methodologically, they demonstrate the powerful potential of modern technologies. Therefore, future studies on L2 listening comprehension can benefit considerably from these technologies.

## Conclusion

With regard to the causal relationship between lexical and syntactic knowledge and L2 listening, each study reviewed earlier has approached the issue by focusing on one particular combination of features, contributing to various degrees of relevance. As Shadish (2010) sees it, any single study sheds a little light on the nature of the causal relationship, but multiple studies on the same question are needed to find out which features are irrelevant to the causal knowledge and which are central. This study is just such an attempt. Built upon earlier studies, it helps find out the key features in lexical and syntactic knowledge that contribute to L2 listening. Using lexical and syntactic measures with similar task characteristics in terms of the visual/auditory, receptive/productive, and contextualized/decontextualized dichotomies, the study has replicated the findings in earlier studies that used similar relevant measures. The results showed that when lexical and syntactic knowledge is measured in auditory receptive tasks contextualized in natural discourse, both measures have unique contributions to L2 listening. The key message from these results is that research instruments should be designed to validly represent constructs if practical research is to yield consistent findings that agree with theory and with each other.

## Data Availability Statement

The raw data supporting the conclusions of this article will be made available by the author, without undue reservation, to any qualified researcher.

## Ethics Statement

Ethical review and approval was not required for the study on human participants in accordance with the local legislation and institutional requirements. Written informed consent for participation was not required for this study in accordance with the national legislation and the institutional requirements.

## Author Contributions

The author confirms being the sole contributor of this work and has approved it for publication.

## Conflict of Interest

The author declares that the research was conducted in the absence of any commercial or financial relationships that could be construed as a potential conflict of interest.

## Footnotes

1 Cheng and Matthews (2018) called their third measure productive because participants had to respond in words. However, the core construct of this task was word recognition, which is receptive in nature. Responding in words does not change the receptive nature of partial dictation as a listening task (Cai, 2013).

2 https://www.ielts.org/ielts-for-organisations/ielts-scoring-in-detail, retrieved as of Nov. 16, 2019.

3 https://www.ielts.org/teaching-and-research/test-taker-performance, retrieved as of Nov. 16, 2019.

4 https://www.ielts.org/en-us/about-the-test/test-format, retrieved as of Nov. 16, 2019.

## Appendix

**Table:** List of phrases in the syntactical scale.

No.	Phrase	Syntactic features
1	1.6 or anything	Ellipsis (“1.6” = 1.6 liters) Noun conjunction (“or”)
2	got one in	Phrase structure (verb + pronoun + adverb particle)
3	with the engine	Phrase structure (preposition + article + noun)
4	go for that	Phrasal verb (“go for”) Pronoun (“that”)
5	is that	Predicate after a parenthesis
6	what you do	Subordination Clause structure (“what” + clause)
7	if this process	Subordination Initial string of a clause Noun phrase as subject (“this process”)
8	for this process	Clause structure (it is + adjective + for someone to do something)
9	if possible	Parenthesis Ellipsis (if… is possible)
10	If you decide	Subordination Initial string of a clause
11	to move on	Cohesive device Phrasal verb (“move on”)
12	our student body	Compound noun
13	the better	Special structure (“The earlier… the better…”)
14	What if	Question beginning
15	the very latest	Emphatic expression
